# Genetic Deletion of Zebrafish Rab28 Causes Defective Outer Segment Shedding, but Not Retinal Degeneration

**DOI:** 10.3389/fcell.2020.00136

**Published:** 2020-03-17

**Authors:** Stephen P. Carter, Ailís L. Moran, David Matallanas, Gavin J. McManus, Oliver E. Blacque, Breandán N. Kennedy

**Affiliations:** ^1^UCD School of Biomolecular and Biomedical Science, University College Dublin, Dublin, Ireland; ^2^UCD Conway Institute, University College Dublin, Dublin, Ireland; ^3^Systems Biology Ireland, University College Dublin, Dublin, Ireland; ^4^School of Biochemistry and Immunology, Microscopy Facility, Trinity Biomedical Sciences Institute, Trinity College Dublin, Dublin, Ireland

**Keywords:** zebrafish, cilia, retinal degeneration, visual function, photoreceptor, small G protein, outer segment

## Abstract

The photoreceptor outer segment is the canonical example of a modified and highly specialized cilium, with an expanded membrane surface area in the form of disks or lamellae for efficient light detection. Many ciliary proteins are essential for normal photoreceptor function and cilium dysfunction often results in retinal degeneration leading to impaired vision. Herein, we investigate the function and localization of the ciliary G-protein RAB28 in zebrafish cone photoreceptors. CRISPR-Cas9 generated *rab28* mutant zebrafish display significantly reduced shed outer segment material/phagosomes in the RPE at 1 month post fertilization (mpf), but otherwise normal visual function up to 21 dpf and retinal structure up to 12 mpf. Cone photoreceptor-specific transgenic reporter lines show Rab28 localizes almost exclusively to outer segments, independently of GTP/GDP nucleotide binding. Co-immunoprecipitation analysis demonstrates tagged Rab28 interacts with components of the phototransduction cascade, including opsins, phosphodiesterase 6C and guanylate cyclase 2D. Our data shed light on RAB28 function in cones and provide a model for RAB28-associated cone-rod dystrophy.

## Introduction

The photoreceptor outer segment (OS) is an elaborate membranous organelle which functions in the detection of light stimuli and their conversion to electrical signals via phototransduction (Fain et al., 2010; Goldberg et al., 2016). Outer segments are modified primary cilia and as such the molecular machinery which regulates transport and signaling within cilia is also essential for OS formation and function (Wheway et al., 2014). Furthermore, blindness due to photoreceptor degeneration (PRD) is a common phenotype of genetic diseases known as ciliopathies, characterized by ciliary dysfunction (Waters and Beales, 2011; Bujakowska et al., 2017).

Photoreceptor OS are composed of flattened, closed disks surrounded by an outer membrane in the case of rods, and open lamellae in cones. New disks/lamellae form at the base of the OS as ciliary ectosomes (Ding et al., 2015; Salinas et al., 2017), which expand via Arp2/3-regulated actin polymerization (Spencer et al., 2019) and gradually migrate upwards as the oldest disks/lamellae at the OS tip are shed and phagocytosed daily by the retinal pigment epithelium (RPE). OS shedding is integral to photoreceptor health and survival: as the OS are exposed to high levels of light, the oldest disks/lamellae accumulate photo-oxidatively damaged compounds (Kevany and Palczewski, 2010). Despite its essential role in photoreceptor biology, the molecular machinery which regulates OS shedding in photoreceptors is poorly described. Early studies identified species-specific differences in OS shedding regulation; for example, frog (*Rana pipiens*) photoreceptors require light to initiate shedding and display limited shedding in the dark (Basinger et al., 1976), while rat rod photoreceptors shed in a circadian manner, with minimal effect from light/dark conditions (LaVail, 1976). In the intervening years, some pathways involved in phagocytosis and subsequent degradation of shed disks in the RPE were elucidated (Bosch et al., 1993; Gibbs et al., 2003; Law et al., 2009; Jiang et al., 2015). More recently, genes important for OS shedding/phagocytosis were identified in zebrafish, including ceramide kinase-like (Cerkl) (Yu et al., 2017) and the ciliary kinesin Kif17 (Lewis et al., 2018). Recently, knockout of the small GTPase RAB28 was shown to result in impaired shedding and/or phagocytosis of material from the tips of mouse cones, but not rods (Ying et al., 2018). Failure to shed old lamellae led to the accumulation of membranous material at cone tips and eventual degeneration and death of the cones, followed by rods. In humans, *RAB28* null and hypomorphic alleles cause autosomal recessive cone-rod dystrophy (arCRD) (Roosing et al., 2013; Riveiro-Álvarez et al., 2015; Lee et al., 2017). To our knowledge, this is the only example of inherited PRD arising exclusively from a disorder of cone OS (COS) shedding.

In *C. elegans*, we previously demonstrated that RAB28 is an IFT and BBSome-associated ciliary protein (Jensen et al., 2016), which regulates extracellular vesicle biogenesis in a subset of ciliated neurons (Akella et al., 2020). Here, we generate zebrafish *rab28* knockout and transgenic reporter models to investigate the localization, function, GTP/GDP nucleotide regulation, and interactome of RAB28 in cone photoreceptors. Localization of RAB28 to the OS is partially dependent on GTP/GDP-binding, overexpression of GTP-preferring RAB28 in cones results in subtle visual behavior defects and RAB28 biochemically associates with components of the phototransduction cascade, as well as vesicle trafficking proteins. Significantly, *rab28* null zebrafish display a 40–50% reduction in OS shedding as early as 15 days post fertilization (dpf), but without evidence of retinal degeneration up to 12 mpf.

## Materials and Methods

### Zebrafish Strains and Maintenance

Zebrafish larvae from 0 to 5 days post fertilization (dpf) were cultured in Petri dishes of E2 medium (0.137M NaCl, 5.4 mM KCl, 5.5 mM Na_2_HPO_4_, 0.44 mM KH_2_PO_4_, 1.3 mM CaCl_2_, 1.0 mM MgSO_4_ and 4.2 mM NaHCO_3_, conductivity ∼1500 μS, pH 7.2) at 27°C on a 14 h/10 h light–dark cycle.

Adult zebrafish were housed in 1.4, 2.8, or 9.5 L tanks in system water and maintained at a temperature of 27°C on a 14 h/10 h light–dark cycle. The UCD facility environmental parameters are reported at Crowley et al. (2019). Juvenile fish were fed an increasingly complex, specialized diet (Special Diet Services) and gradually transferred to a diet of mainly brine shrimp (Artemia sp.). Zebrafish strains used in this study were:

WT (Tü), *rab-28^*ucd*7^, rab-28^*ucd*8^*, Tg[gnat2:eGFP], Tg[gnat2:eGFP-rab28], Tg[gnat2:eGFP-rab28^*Q*72*L*^] and Tg[gnat2:eGFP-rab28^*T*26*N*^].

### Ethics Statement

All animal experiments were conducted with the approval of the UCD Animal Research Ethics Committee (AREC-Kennedy) and the Health Products Regulatory Authority (Project authorization AE18982/P062). All experiments were performed in accordance with relevant guidelines and regulations.

### Generation of *rab28* Mutant Zebrafish

sgRNAs were designed using the ZiFiT Targeter (v4.2) online tool. Several sgRNAs were designed against the zebrafish *rab28* cDNA sequence. The sgRNA against exon 2 of *rab28* was chosen as there was sufficient genomic sequence data to facilitate genotyping. sgRNAs were cloned into the pDR274 vector (Addgene) following a previously described protocol (Hwang et al., 2013). CRISPR mutants were generated by microinjection of Cas9-sgRNA ribonucleoprotein particles (RNPs) into one-cell stage WT embryos (Cas9 protein was acquired from Integrated DNA Technologies). P_0_ injected fish were raised to adulthood and screened for germline transmission of potential *rab28* null alleles. These were outcrossed to a WT line and the subsequent heterozygous F_1_ fish raised and in-crossed to generate homozygous *rab28^–/–^* larvae.

### Zebrafish Transgenesis

Transgenic zebrafish expressing eGFP-Rab28 in cone photoreceptors were generated by microinjection of plasmids containing a Tol2-gnat2:eGFP-rab28(cDNA)-Tol2 construct, together with Tol2 transposase mRNA. Plasmids were generated by MultiSite Gateway cloning using the Tol2kit and following a previously described protocol (Kwan et al., 2007). The *gnat2* promoter was cloned previously (Kennedy et al., 2007). The zebrafish *rab28* cDNA clone was acquired from the Zebrafish Gene Collection (IMAGE ID: 2643307). The T26N (GDP-preferring) and Q72L (GTP-preferring) mutants of RAB28 were generated by site-directed mutagenesis of the cDNA. Injected embryos were treated with 75 μM phenylthiourea (PTU, Sigma) diluted in embryo medium to suppress melanogenesis and screened for expression of eGFP at 5 dpf. Those larvae positive for eGFP were raised to adulthood and outcrossed to a WT line to generate heterozygous F_1_ transgenic carriers.

### Molecular Biology

sgRNAs and Tol2 transposase mRNA were generated by *in vitro* transcription using the MEGAshortscript and mMessage mMachine SP6 kits (Invitrogen), respectively, following the manufacturer’s protocol. RNA was purified by LiCl precipitation. Genotyping PCRs were performed using MyTaq Red DNA polymerase (Bioline) for 30 cycles with a 72°C extension temperature. For RT-PCR, 5 dpf zebrafish larvae were placed in RNAlater and stored at 4°C overnight. Larvae were homogenized by aspiration through a needle and syringe and RNA was extracted from the resulting lysate using the mirVana RNA isolation kit (Life Technologies), following the manufacturer’s protocol. RNA was subsequently purified and concentrated by ethanol precipitation. cDNA was generated from the isolated RNA using the RevertAid cDNA synthesis kit (Thermo Fisher). This cDNA was then used as template DNA for subsequent PCR reactions.

Primers used in genotyping and generating transgenic constructs are provided in Table 1. Underlined nucleotides indicate mutated positions.

**TABLE 1:** Sequences of primers used in this study.

Primer name	Sequence (5′–3′)
*rab28 fwd* (genotyping)	CGTCTCTCGCCATCCGCTTCGC
*rab28 rev* (genotyping)	GTGTACATATGTGTTTACCTGTGAG
*rab28_attB2r_fwd*	GGGGACAGCTTTCTTGTACAAAGTGGGATCGGACTCCGAGGAGGAG
*rab28_attB3_rev*	GGGGACAACTTTGTATAATAAAGTTGTCACACACAGATCTCAGACGG
*rab28_SDM_Q72L_fwd*	GTCTGGGACATCGGTGGACTGACTATTGGAGGAAAAATG
*rab28_SDM_Q72L_rev*	CATTTTTCCTCCAATAGTCAGTCCACCGATGTCCCAGAC
*rab28_SDM_T26N_fwd*	GACGGAGCGTCAGGGAAGAACTCTCTCGCCATCCGCTTC
*rab28_SDM_T26N_rev*	GAAGCGGATGGCGAGAGAGTTCTTCCCTGACGCTCCGTC

### Behavioral Assays

The optokinetic response (OKR) assay was performed by immobilizing 5 dpf larvae in 9% methylcellulose in a 55 mm Petri dish. The dish was placed inside a rotating drum with a black and white striped pattern on the inside with 18 degrees per stripe, contrast 99%. The drum was rotated clockwise and anticlockwise for 30 s each, at a speed of 18–21 rpm, during which time the number of eye movements (saccades) of the fish were manually recorded using a stereomicroscope. At least 30 larvae per transgenic strain, 32 mutants and 98 siblings were analyzed across three experimental replicates. The visual motor response (VMR) assay was performed using the ZebraBox^®^ recording chamber (ViewPoint). 5 dpf larvae were placed in individual wells of a 96 well polystyrene plate in 600 μl of embryo medium, which was placed in the recording chamber. Locomotor activity of the larvae in response to changing light conditions was recorded using an infrared camera. Data analysis was performed as previously described (Deeti et al., 2014). All OKR and VMR experiments were performed during the afternoon, to avoid variations due to diurnal rhythms (Huang et al., 2018). At least 64 larvae per transgenic strain, 32 mutants and 49 siblings were analyzed.

### Protein Extraction and Immunoblotting

5 dpf zebrafish larvae were killed on ice and eyes dissected in a solution of 5 mM NaCl with protease inhibitor cocktail tablets (Roche). Eyes were either snap frozen in liquid nitrogen and stored at −80°C or immediately lysed. Protein concentration was estimated by Bradford assay to ensure equivalence between samples. Proteins were separated on a 0.75 mm 12% Bis/Tris acrylamide SDS-PAGE resolving gel and transferred to nitrocellulose membranes. Membranes were blocked in 5% skim milk-PBST for 1 h at room temperature and subsequently probed with primary antibodies at 4°C overnight, followed by secondary antibodies. Primary antibodies used in this study were anti-GFP (1:500, Santa Cruz Biotechnology) and anti-PDE6D (1:500, Abcam). Secondary antibodies were HRP-conjugated anti-rabbit or HRP-conjugated anti-mouse (both 1:2000, Cell Signalling Technology). Blots were performed *n* = 2 for each primary antibody.

### Immunoprecipitation

Immunoprecipitations were performed on 5 dpf larval eyes (100 per replicate) lysed in IP lysis buffer [50 mM Tris HCl, pH 7.5, 150 mM NaCl, 10 mM MgCl_2_, 1% NP-40, 1 mM DTT, 1 mM PMSF, 2 mM Na_3_VO_4_ and protease inhibitor cocktail (Roche), 1 tablet per 10 ml]. Tissue was disrupted by aspiration through a needle and syringe, followed by a 20 min incubation on a tube rotator (Stuart) at 4°C. Lysates were cleared by centrifugation at 20,000 × *g* for 15 min, the supernatant was loaded onto GFP-Trap beads (Chromotek) and incubated on a rotor at 4°C for 2 h or overnight. For nucleotide addition, the lysate was split into three equal volumes prior to bead loading and either GTPγS or GDP (Sigma-Aldrich) was added at a final concentration of 1 mM and incubated for 20 min on ice, with the third tube serving as a negative control. Following this the beads were pelleted by centrifugation at 2500 *g* and washed three times with lysis buffer. For immunoblotting, proteins were eluted from the beads with SDS sample buffer followed by boiling at 95°C for 5 min. For mass spectrometry, a previously described protocol was followed (Turriziani et al., 2014). Briefly, proteins were trypsinised on the beads in 60 μl of Buffer I (2M urea, 50 mM Tris-HCl pH 7.5, 5 μg/ml Trypsin [modified sequencing-grade trypsin; Promega]) for 30 min at 37°C in a thermomixer, shaking at 700 rpm. Samples were briefly centrifuged and supernatants transferred to clean Eppendorf tubes. The beads were then incubated in 50 μl Buffer II (2M urea, 50 mM Tris-HCl pH 7.5, 1 mM DTT) for 1 h at 37°C, shaking at 700 rpm in a thermomixer. Samples were again briefly centrifuged, and the supernatants from Buffers I and II pooled and left to continue trypsin digestion overnight at room temperature.

### Mass Spectrometry

Samples from the overnight digest were alkylated by addition of 20 μl iodoacetamide (5 mg/ml) and incubation for 30 min in the dark. 1 μl 100% trifluoroacetic acid (TFA) was added to the samples to stop the reaction and samples were then loaded onto equilibrated C18 StageTips containing octadecyl C18 disks (Sigma) (Turriziani et al., 2014). Briefly, a small disk of Empore material 3M was inserted into a pipette tip, preparing a single tip for each sample. Tips were activated and equilibrated by washing through 50 μl of 50% acetonitrile (AcN) – 0.1% TFA solution followed by 50 μl of 1% TFA solution, using a syringe to pass liquid through the pipette tips. Once added to StageTips, samples were desalted by washing twice with 50 μl of 1% TFA solution. Peptides were then eluted into clean Eppendorf tubes using 2 × 25 μl 50% AcN – 0.1% TFA solution. The final eluates were concentrated in a CentriVap concentrator (Labconco, United States) and re-suspended in 12 μl 0.1% TFA solution, ready for analysis by mass spectrometry (Turriziani et al., 2014). Peptides were analyzed on a quadrupole Orbitrap (Q-Exactive, Thermo Scientific) mass spectrometer equipped with a reversed-phase NanoLC UltiMate 3000 HPLC system (Thermo Scientific). Three biological and two technical replicates were performed per transgenic line. To identify peptides and proteins, MS/MS spectra were matched to the UniProt Danio rerio database. LFQ intensities were subsequently analyzed using Perseus (v1.6.1.3) (Tyanova et al., 2016). Protein identifications were filtered to eliminate the identifications from the reverse database and common contaminants. Data was log_2_ transformed and *t*-test comparison of fractions carried out. Gene ontology terms were identified and visualized by submitting identified gene lists to the PANTHER database (Thomas et al., 2003). The mass spectrometry proteomics data have been deposited to the ProteomeXchange Consortium via the PRIDE (Perez-Riverol et al., 2019) partner repository with the dataset identifier PXD017523.

### Fluorescence Microscopy

Zebrafish were euthanized with tricaine methanesulfonate, fixed in 4% PFA overnight at 4°C and subsequently washed with PBS, cryoprotected in a sucrose gradient ascending series and finally embedded in OCT (VWR). 10 μm thick frozen sections were cut on a Microm HM 505 E cryostat and mounted on Superfrost Plus slides (Thermo Fisher). Sections were stained with the following primary antibodies: rat anti-GFP (1:500, Santa Cruz Biotechnology), rabbit anti-UV opsin [1:250, a gift from David Hyde (Vihtelic et al., 1999)] or rabbit anti-cone transducin α [1:50 a gift from Susan Brockerhoff (Brockerhoff et al., 2003)]. Secondary antibodies were Alexa 488 or Alexa 567-conjugated (1:500, Thermo Fisher), respectively. Following antibody incubation, sections were stained with DAPI. Slides were then mounted with Mowiol^®^ (Merck) and cover-slipped. For larvae, a total of 14, 24 and 25 individuals were imaged for WT, Q72L and T26N eGFP-Rab28 reporters, respectively. For adults, a total of 13, 11, and 11 retinas across at least six individuals were imaged for each reporter.

For disk shedding analysis, fish were euthanized and fixed at the peak shedding times, i.e., 4 h post lights-on and 4 h post lights-off (Lewis et al., 2018). For analysis of *rab28* mutants and siblings, anti-UV opsin, anti-red opsin and anti-cone transducin α antibodies were used to label phagosomes. 13 and 11 retinal z-projections from at least three individuals were imaged for mutant and siblings, respectively. For analysis of eGFP-Rab28 transgenics, eGFP fluorescence was used to identify phagosomes. 17, 17, and 13 retinal z-projections from at least three individuals were imaged for each reporter.

Immunostained zebrafish retinal sections were imaged on an inverted Zeiss LSM 510 Meta confocal laser scanning microscope. High-resolution images of eGFP-Rab28 localization were taken with an Olympus FLUOVIEW FV3000 confocal microscope for 5 dpf retinas and with a Leica TCS SP8 X for 1 mpf retinas (resolution 120–200 nm). Images were deconvolved using Huygens Professional software (Scientific Volume Imaging B.V.) All image analysis was performed using Fiji (Schindelin et al., 2012).

### Transmitted Light Microscopy

Zebrafish were euthanized with tricaine methanesulfonate and the eyes enucleated and fixed overnight at 4°C in 2% PFA and 2.5% glutaraldehyde in 0.1M Sorenson phosphate buffer pH 7.3. Samples were post-fixed in 1% osmium tetroxide and dehydrated in a gradient ascending series of ethanol concentrations prior to embedding in Epon 812 resin overnight. 1 μm sections were prepared using a Leica EM UC6 microtome and glass knife, mounted on glass slides and stained with toluidine blue. The appearance of the lens core was used as a landmark to ensure similarity of samples in imaging and measuring. Sections were imaged with a Nikon Eclipse 80i upright microscope equipped with a Canon EOS 600D camera.

### Transmission Electron Microscopy

Zebrafish eyes were embedded for TEM using the same protocol for light microscopy. 90 nm sections were cut on a Leica EM UC6 microtome, mounted on copper grids and post-stained with 2% uranyl acetate and 3% lead citrate. Imaging was performed on an FEI Tecnai 120 electron microscope. For ultrastructural analysis, one retina from each strain (*rab28* mutant, sibling and the three eGFP-Rab28 transgenics), was imaged. For phagosome analysis by TEM, phagosomes were manually counted and the density calculated as phagosomes per μm of RPE. Three mutants and siblings were sectioned and imaged.

### Statistics and Data Analysis

Statistical analysis of all data was performed using GraphPad Prism (v5). CRISPR/Cas9 *rab28* knockout OKR data was analyzed by unpaired *t*-test, transgenic OKRs were analyzed by one-way ANOVA. In the VMR assay, activity was taken as the sum of medium (middur) and high (burdur) activity levels, as measured by the ZebraBox, and plotted against time. Activity traces show the average of multiple individuals over the time period. For graphs of peak activity, the average of 5 s of activity for individual larvae after a light change was plotted. For mass spectrometry, data derived from MaxQuant were uploaded to Perseus (v1.6.1.3). Reverse, contaminant or identified-by-site protein IDs were removed. LFQ intensity value was log_2_ transformed and significantly enriched proteins identified by two sample *t*-test, comparing each eGFP-Rab28 variant to the eGFP only control.

## Results

### Zebrafish *rab28* and CRISPR Mutagenesis

**FIGURE 1:**
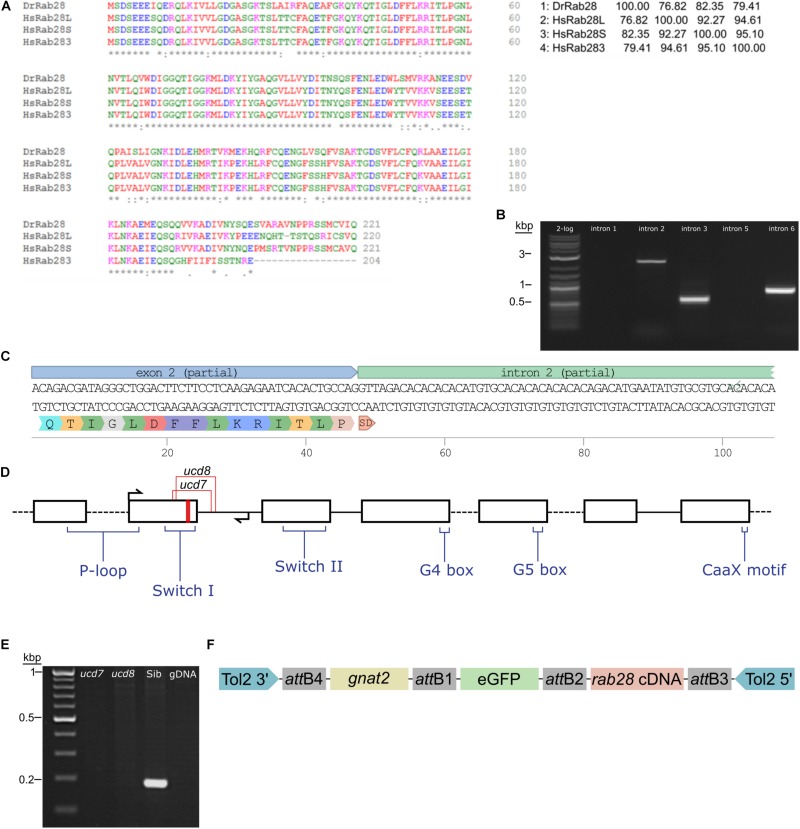
Sequencing of the zebrafish *rab28* gene, CRISPR mutagenesis and Tol2 transgenesis. **(A)** Multiple sequence alignment of zebrafish Rab28 protein sequence, predicted from mRNA, with that of the three known human RAB28 protein isoforms. The percentage protein identity is shown in a matrix table. **(B)** Representative gel showing PCR amplification of predicted *rab28* introns 2, 3, and 6. **(C)** DNA sequencing reveals the exon–intron boundary at exon 2 of zebrafish *rab28* and the predicted translated sequence encoded by exon 2. SD: splice-donor site. **(D)** Schematic of predicted *rab28* gene structure. Black boxes represent exons and connecting lines represent introns. Red stripe indicates location of sgRNA target site used to generate CRISPR mutants, arrows indicate genotyping primer positions, coding positions for critical Rab GTPase motifs are highlighted and the location of *ucd7* and *ucd8* indels indicated. **(E)** Example RT-PCR gel showing the absence of correctly spliced *rab28* cDNA between exons 2 and 4 in homozygous *ucd7* and *ucd8* larvae, which is present in WT siblings. **(F)** Schematic of the construct used to generate eGFP-Rab28 transgenic zebrafish. Expression is driven by the *gnat2* (cone transducin alpha) promoter (yellow box). *att*: Gateway att sites, Tol2 3′ and 5′: transposon inverted repeats.

We initiated CRISPR knockouts by characterizing the zebrafish ortholog of *rab28*. While the locus and genomic sequence were unknown, a sequenced mRNA transcript was reported (RefSeq: NM_199752.1). Human RAB28 has three splice isoforms (Roosing et al., 2013), whereas there is only one known zebrafish isoform. It most closely matches the human RAB28S isoform in sequence (Figure 1A). Due to poor annotation of the zebrafish *rab28* locus, it was necessary to obtain genomic DNA sequence to accurately design CRISPR sgRNAs to target the gene. To design primers for sequencing it was necessary to estimate the exon–intron structure of zebrafish *rab28*. Primers were designed to each potential exon of *rab28* to amplify the intervening introns. A product was successfully amplified in three reactions, the others failing either because the prediction of exon placement was incorrect or the introns were too large to amplify by PCR (Figure 1B). The amplified products were subcloned and DNA sequencing confirmed the predicted positions of exons 2 and 3 (Figures 1C,D). This sequence information facilitated the design of sgRNAs and genotyping strategies for the *rab28* KO lines. Cas9-rab28exon2 sgRNA ribonucleoprotein particles (RNPs) were injected into one-cell stage embryos, which were subsequently raised to adulthood. Adult P_0_ fish were genotyped for the presence of mutant *rab28* alleles by PCR and outcrossed to detect germline transmission. Two *rab28* mutant lines were generated, *rab28^*ucd*7^* and *rab28^*ucd*8^*, a 40 bp deletion and a 14 bp insertion or a 65 bp deletion and an 8 bp insertion, respectively. Both alleles disrupt part of exon 2 coding sequence and the intron 2 donor site. Retention of intron 2 is predicted to lead to truncation of Rab28 due to the presence of in-frame stop codons and therefore the loss of several functional motifs from the translated protein (Figure 1D). RT-PCR of RNA from homozygous knockout larvae shows an absence of the correctly spliced transcript (Figure 1E), suggesting that the transcript is degraded by nonsense-mediated decay. Zebrafish homozygous for each of these alleles were subsequently used in phenotypic analyses.

In order to assess localization, function and protein-protein interactions of Rab28, we generated a transgenic fish line expressing an eGFP-Rab28 construct in cone photoreceptors (Figure 1F). We also generated two further transgenic lines, one harboring the (predicted GTP-preferring) Q72L mutation and another the (predicted GDP-preferring) T26N mutation. It should be noted that the TN mutation commonly used to ‘GDP-lock’ Rabs lowers the affinity for guanine nucleotides generally (Lee et al., 2009), so our T26N mutant may mimic the nucleotide empty state.

### *rab28* Mutant Zebrafish Have Normal Visual Function at 5 dpf

**FIGURE 2:**
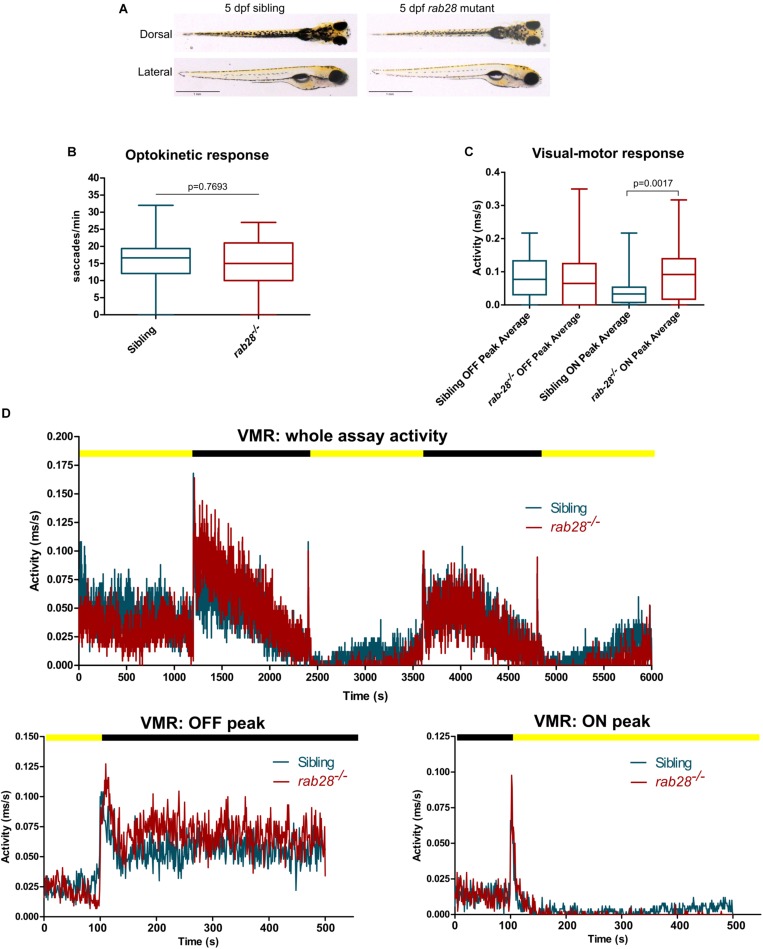
*rab28* knockout zebrafish have subtle defects in visual behavior at 5 dpf. **(A)** Representative images of *rab28* knockout and sibling zebrafish larvae at 5 dpf. Gross morphology is indistinguishable between knockouts and siblings. **(B)** Box and whisker plot of the optokinetic response (OKR) of *rab28* knockout larvae versus sibling larvae at 5 dpf. OKRs are not significantly different. Box extremities represent 1st and 3rd quartiles; whiskers are maximum and minimum values. *p*-Value derived from unpaired *t-*test. OKR data are from 32 mutants and 98 siblings, across three experimental replicates. **(C)** Box and whisker plot of 5 dpf larval activity during the visual-motor response (VMR) assay. OFF peak activity is identical between *rab28* knockouts and siblings, albeit *rab28* knockouts have an average 51% higher ON peak activity. Data are from three independent replicates and are the average of 5 s of activity following light changes. Box extremities represent 1st and 3rd quartiles; whiskers are maximum and minimum values. *p*-Value derived from unpaired *t*-test. **(D)** Activity traces showing 5 dpf larval activity over the course of an entire VMR assay (100 min), as well as separate graphs showing activity 100 s before and 400 s after OFF and ON peaks, respectively. Black and yellow bars indicate dark and light conditions, respectively. VMR data are from 32 mutants and 49 siblings, across three experimental replicates.

Compared to sibling controls, *rab28^–/–^* larvae display normal development and gross morphology at 5 dpf (Figure 2A). To assess visual function in *rab28^–/–^* zebrafish, we utilized two behavioral assays: the optokinetic response (OKR) and visual-motor response (VMR). Homozygous mutant larvae and control (+ / + and ±) siblings for both CRISPR alleles were assessed at 5 dpf, between the hours of 12 and 4 pm. The OKR of *rab28^–/–^* larvae was not different to control siblings at 5 dpf, under the conditions tested (Figure 2B). To investigate the possibility of a reduction in visual function at a later age, we performed OKR assays on 21 dpf *rab28* mutants and controls (Supplementary Figure 1A). As at 5 dpf, the OKR of *rab28* knockouts was not significantly different from siblings at 21 dpf (*p* = 0.5224), under the test conditions. In the VMR assay, at 5 dpf, the OFF peak activity between siblings and mutants is identical, whereas *rab28* mutants display a 51% increased average ON peak activity (Figure 2C; *p* = 0.0017). The overall activity traces and the OFF and ON peak traces (100 s before and 400 s after a light change) highlight *rab28* mutants with slightly elevated activity in the dark, but reduced activity in light conditions, compared to sibling controls (Figure 2D). These data show that, in zebrafish at 5 dpf, *rab28* knockout results in subtle effects on visual behavior compared to WT.

### *rab28* Mutants Have Normal Retinal Histology and Ultrastructure Up to 12 mpf

**FIGURE 3:**
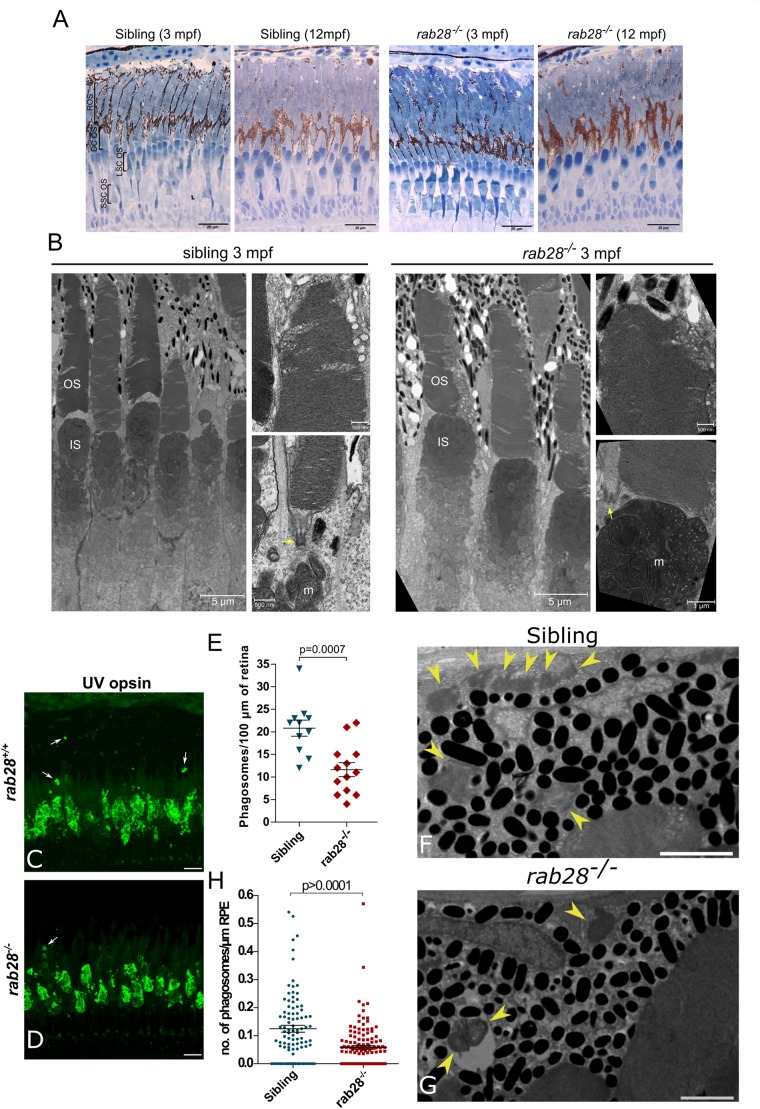
*rab28* knockout zebrafish have reduced outer segment shedding, but normal retinal histology and ultrastructure. **(A)** Representative images of retinal histology in 3 and 12 mpf zebrafish, showing views of the photoreceptor layer in the central and peripheral retina in *rab28^*ucd*8^* mutants and siblings. The overall structure and composition of the photoreceptor layer is grossly normal in both. Scale bars 20 μm **(B)** Representative transmission electron micrographs of 3 mpf zebrafish *rab28^*ucd*7^* knockout and sibling retinas. Low magnification images show several cone photoreceptors, while high magnification images show examples of OS base and tips. Yellow arrows indicate ciliary basal body. OS: outer segment; IS: inner segment; m: mitochondria. Low magnification image scale bars 5 μm, high magnification scale bars 500 nm. **(C,D)** Confocal z-projections of *rab28* mutant and sibling control retinas at 1 mpf, stained for UV opsin to label phagosomes (white arrows). Samples were collected 4 h after lights on. Scale bars 10 μm **(E)** Scatter plots of phagosome density in *rab28* mutant and sibling retinas. Data are derived cryosections immunostained for UV and red opsins and cone transducin α. *p*-Value is derived from *t*-test. Error bars show SEM. Data are from 13 and 11 retinal z-projections from at least three individuals for mutants and siblings, respectively. **(F,G)** Representative TEM of RPE phagosomes in 15 dpf *rab28* mutants and sibling controls. Yellow arrows indicate phagosomes. Samples were collected 4 h after lights on. Scale bars 2 μm. **(H)** Scatter plots of phagosome density in 15 dpf *rab28* mutant and sibling retinas, derived from TEM. *p*-Value is derived from *t*-test. Error bars show SEM. Data are from three sibling and three mutant individuals.

The absence of visual behavior deficits in larval and juvenile *rab28^–/–^* fish led us to investigate the possibility of a slow-onset, progressive retinal degeneration, as observed in other zebrafish models, such as *eys* and *rpgrip1* (Yu et al., 2016; Raghupathy et al., 2017). Thus, homozygous *rab28^–/–^* larvae were raised to adulthood and retinal histology assessed. At 3 mpf, the retina of a *rab28^–/–^* had equivalent retinal lamination to a sibling control and the photoreceptor layer contained all five photoreceptor cell types in their normal distribution and abundance (Figure 3A). We then assessed retinal histology in a 12 mpf sibling and *rab28* mutant and again found mutant retinas to be healthy, with no evidence of degeneration. To investigate potential ultrastructural defects, TEM was performed on 3 mpf retinas. Photoreceptor ultrastructure was similar between sibling and mutant at 3 mpf. Both genotypes show normal basal body positioning, while cone lamellae in *rab28^–/–^* fish display normal organization and alignment (Figure 3B). Therefore, loss of *rab28* is not associated with pronounced retinal structure degeneration in zebrafish, up to 12 mpf.

### *rab28* Mutant Zebrafish Have Reduced Shedding of Cone OS Disks

Defective cone outer segment shedding was investigated in *rab28^–/–^* zebrafish, as this phenotype was recently reported for *rab28*^–/^*^–^* mice (Ying et al., 2018). Unlike mice, two shedding peaks are reported for zebrafish photoreceptors: one in the morning and one in the evening (Lewis et al., 2018). Both rods and cones are reported to shed at these time points. In *rab28^–/–^* zebrafish, immunofluorescence staining was applied to identify cone OS protein staining (cone opsins and cone transducin alpha) located distal to the tips of the cone outer segments as a surrogate measure of RPE phagosomes containing shed outer segments, as previously reported (Esteve-Rudd et al., 2018; Ying et al., 2018). At 1–2 mpf, the number of cone phagosomes are reduced in *rab28* mutant zebrafish by ∼44% compared to siblings (Figures 3C–E). To validate this finding, we performed TEM on the retinas of 15 dpf *rab28* mutants and siblings and counted the number of RPE phagosomes. Phagosome number was reduced in *rab28* mutants by 53% on average (Figures 3F–H). Our data demonstrate a conserved role for Rab28 in OS shedding in cone photoreceptors.

### eGFP-Rab28 Localization to Cone Outer Segments Is Partially Dependent on GTP/GDP Binding

**FIGURE 4:**
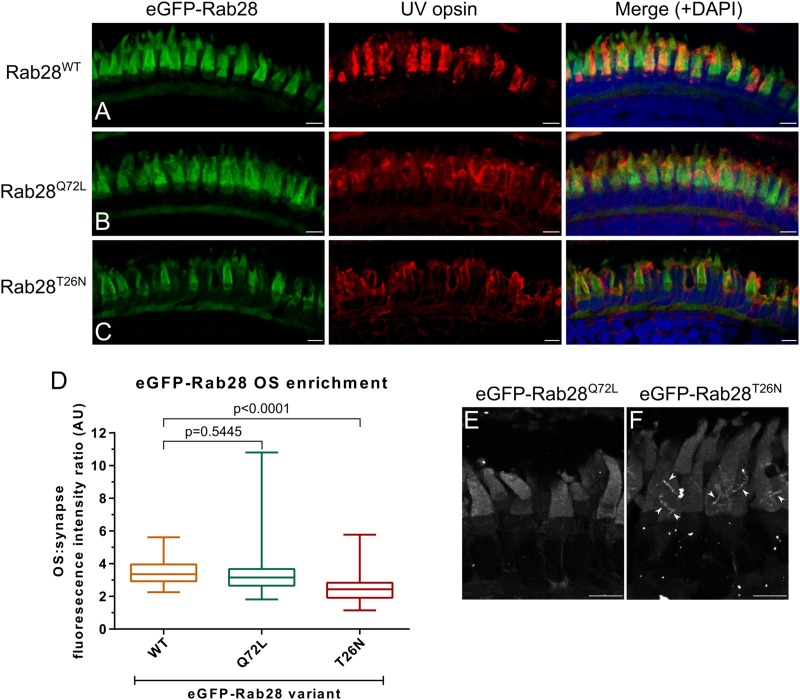
eGFP-Rab28 localization to larval zebrafish cone outer segments is partially dependent on GTP/GDP-binding. **(A–C)** Representative confocal z-projections of eGFP-Rab28 localization in 5 dpf zebrafish cone photoreceptors. The WT, putative GTP-preferring (Q72L) and GDP-preferring (T26N) variants of Rab28 all localize strongly to the outer segments of zebrafish cones, co-localizing with UV opsin labeling. Scale bars 5 μm. For WT, Q72L and T26N eGFP-Rab28 reporters a total of 14, 24 and 25 larvae were imaged, respectively. **(D)** Box and whisker plots of the ratio of eGFP-Rab28 intensity in the OS vs. synaptic region of larval cones. Box extremities represent 1st and 3rd quartiles; whiskers are maximum and minimum values. Data are from 60 cones per transgenic line. One-way ANOVA *p*-value < 0.0001. **(E,F)** Deconvolved, high resolution confocal z-projections of eGFP-Rab28 Q72L and T26N mutant localization in cones of 5 dpf larvae. A discrete localization pattern of the T26N mutant in COS is clearly observed (white arrowheads). Scale bars 4 μm.

GTPase switching between the GTP or GDP-bound conformations is often accompanied by a change in protein localization to another cellular compartment. We previously reported that GTP and GDP-binding variants of *C. elegans* RAB-28 dramatically alter localization in ciliated sensory neurons (Jensen et al., 2016). While RAB28 localizes to murine rod and cone OS (Ying et al., 2018), it is unknown if this is influenced by nucleotide binding. This question was investigated with three eGFP-Rab28 variants expressed in zebrafish cones. Confocal imaging of cryosections from 5 dpf zebrafish revealed eGFP-Rab28 localized almost exclusively to cone OS (Figures 4A–C). However, in the T26N mutant, an average 30% reduction in OS enrichment was observed compared to the WT variant (Figure 4D; *p* < 0.0001), suggesting less efficient targeting of GDP-bound or nucleotide empty eGFP-Rab28 to OS.

**FIGURE 5:**
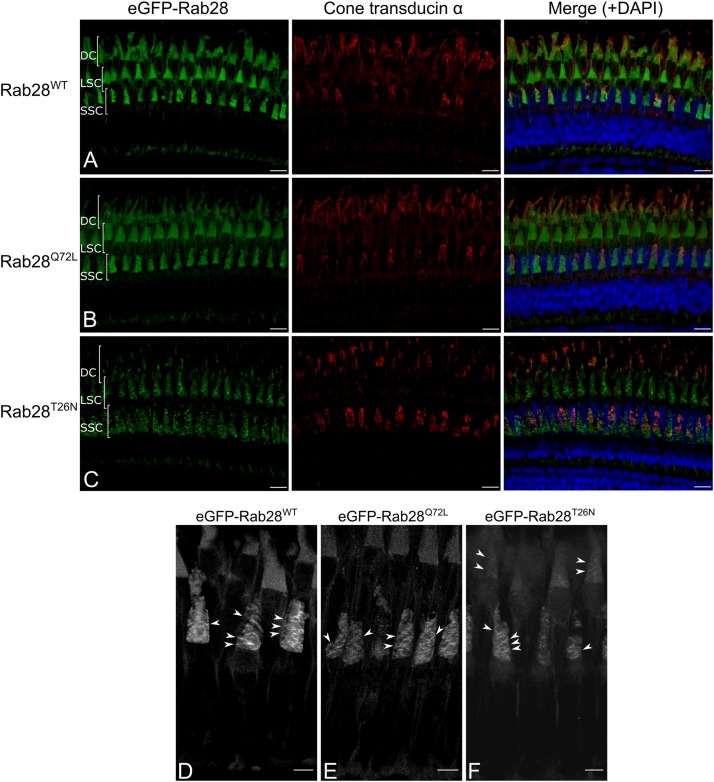
eGFP-Rab28 localization in 1 mpf zebrafish cone photoreceptors. **(A–C)** Representative confocal z-projections of eGFP-Rab28 localization in 1 mpf zebrafish cones. Cone OS are stained with anti-cone transducin alpha antibody. Each tier of photoreceptors is comprised of different classes of cone. DC: double cones; LSC: long single cones; SSC: short single cones. Scale bars 10 μm. For WT, Q72L and T26N eGFP-Rab28 reporters a total of 13, 11 and 11 retinas across at least six individuals were imaged, respectively. **(D–F)** Deconvolved, high resolution confocal z-projections of eGFP-Rab28 WT, Q72L and T26N mutant localization in 1 mpf cones. Arrowheads point to discrete bands present throughout the OS. Scale bars 5 μm.

Intriguingly, the eGFP-Rab28^T26N^ mutant reporter was observed to occasionally localize to discrete, horizontal bands in the OS of some cones (Figures 4E,F). The OS of zebrafish photoreceptors are fully mature by 24 dpf (Branchek and Bremiller, 1984). To investigate whether further photoreceptor development is accompanied by changes in Rab28 localization, retinal sections were imaged from 1 mpf zebrafish. Again, the WT and mutant versions of eGFP-Rab28 were strongly enriched in the OS of all cones (Figures 5A–C). Strikingly, at this time point, discrete banding patterns were observed in all three transgenic lines, in a larger number of photoreceptors and was far more extensive than in larvae, occurring at regular intervals from base to tip of cone OS (Figures 5D–F). In the WT and Q72L mutant reporters, banding appeared largely restricted to the short single (SS) cone population, located in the bottommost row of photoreceptors (Figures 5A,B,D,E). The T26N reporter, by contrast, showed discrete banding in other cone populations (Figures 5C,F), though still primarily in SS cones. Our data show that Rab28 is efficiently targeted to cone OS in a manner only partially dependent on its nucleotide-bound state, where it is organized into discrete segments of the OS, a behavior that appears to be more prominent when in the GDP-bound/nucleotide free state.

### eGFP-Rab28 Transgenic Zebrafish Have Reduced Visual Function at 5 dpf

**FIGURE 6:**
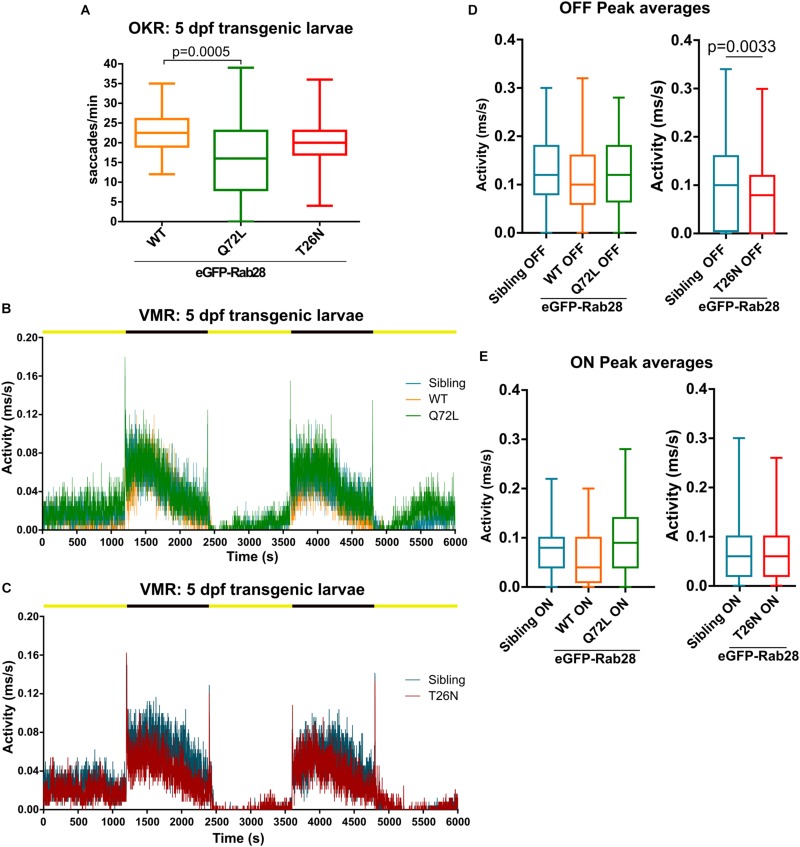
Rab28 transgenic zebrafish have mild to moderate visual defects at 5 dpf. **(A)** Box and whisker plot of optokinetic response (OKR) assay of 5 dpf larvae overexpressing GFP-Rab28 WT, Q72L (GTP-preferring) or T26N (GDP-preferring). Larvae expressing the Q72L variant display greater variability and an overall reduction in OKR scores. Box extremities represent 1st and 3rd quartiles; whiskers are maximum and minimum values. Data are from three independent replicates; at least 30 larvae analyzed per strain over three experimental replicates; *p*-value derived from one-way ANOVA. **(B,C)** Representative activity traces of 5 dpf transgenic and sibling larval activity over the course of the entire VMR assay. Black and yellow bars indicate dark and light conditions, respectively. **(D,E)** Box and whisker plots of the OFF and ON peak activity of 5 dpf transgenic and sibling larvae. Data are from three independent replicates and are the average of 5 s of activity following light changes. At least 64 larvae were analyzed per strain. *p*-Value derived from unpaired *t*-test **(D)**.

We previously demonstrated overexpression of either GTP or GDP-preferring RAB28 induces functional and ultrastructural defects in the cilia and sensory organs of the nematode *C. elegans*. To assess an evolutionarily conserved function of this nucleotide binding domain in the vertebrate retina, we assessed visual function in transgenic eGFP-Rab28 zebrafish larvae (Figures 6A–E and Supplementary Figures 1A–E). In the OKR, 5 dpf transgenic larvae expressing eGFP-Rab28^WT^ displayed normal saccadic eye movements equivalent to non-transgenic fish (18–25/min) (Figure 6A). eGFP-Rab28^Q72L^ larvae, however, had a far greater range of responses and an average 30% reduction in OKR, while the eGFP-Rab28^T26N^ expressing larvae had similar responses to WT (Figure 6A).

By contrast, the VMR assay of eGFP-Rab28^WT^ and eGFP-Rab28^Q72L^ 5 dpf larvae showed similar light and dark activity compared to non-transgenic sibling controls (Figures 6B,D,E and Supplementary Figures 1B,C), while the dark, but not light, activity of eGFP-Rab28^T26N^ larvae was reduced (Figures 6C–E and Supplementary Figures 1D,E). T26N transgenic larvae also had significantly reduced OFF peak activity compared to siblings (Figure 6D). Overall, these data show that transgenic larvae overexpressing eGFP-Rab28 GTP and GDP-preferring mutants display mild to moderate defects in visual function at 5 dpf.

### eGFP-Rab28 Transgenic Zebrafish Have Normal Photoreceptor Ultrastructure at 7 mpf and Normal Outer Segment Shedding

**FIGURE 7:**
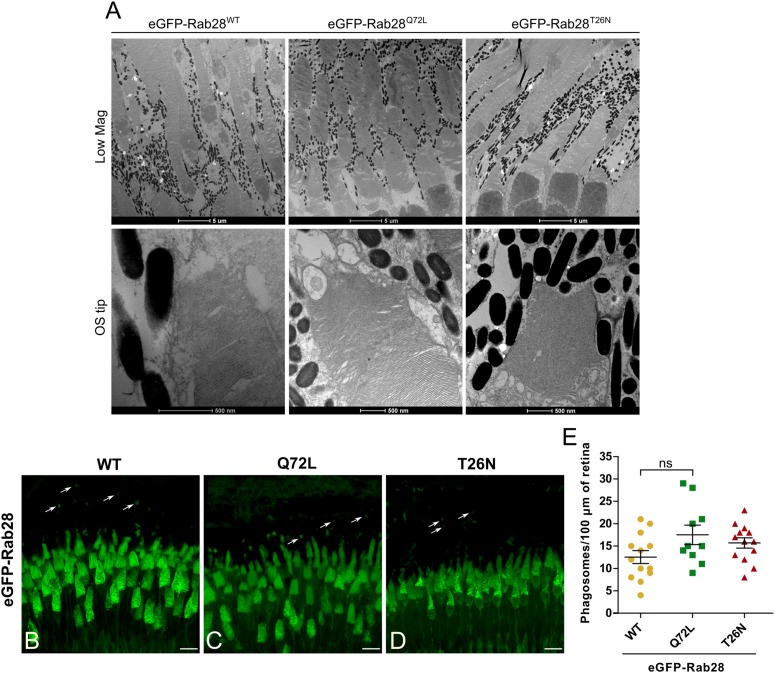
Rab28 transgenic zebrafish have normal retinal ultrastructure and normal outer segment shedding. **(A)** Representative TEM of 7 mpf eGFP-Rab28 transgenic zebrafish. Low magnification images show rows of several cone photoreceptors, while high magnification images show examples of OS tips. Low magnification scale bars 5 μm, high magnification scale bars 500 nm. **(B–D)** Confocal z-projections of 1 mpf retinas of zebrafish expressing eGFP-Rab28 (WT, Q72L, or T26N variants). Phagosomes, labeled with eGFP, are indicated by white arrows. Samples were collected 4 h after lights on. Scale bars 10 μm. **(E)** Scatter plots showing normalized phagosome density in the retinas eGFP-Rab28 transgenic zebrafish. Data are derived from cryosections in which eGFP was used to identify phagosomes. *p*-Value derived from one-way ANOVA. Error bars show SEM. Data are from 17, 17, and 13 retinal z-projections from at least three individuals for WT, Q72L, and T26N variants, respectively.

Given the slight visual behavior defects exhibited by eGFP-Rab28 mutant expressing larvae, we assessed photoreceptor ultrastructure in 7 mpf adults expressing the three different Rab28 reporters. We found that eGFP-Rab28 overexpressing cones had no obvious ultrastructural defects and normal outer segment morphology (Figure 7A). Thus, overexpression of eGFP-Rab28 or its GTP/GDP-preferring mutants does not adversely affect cone ultrastructure up to 7 mpf.

Given our observation of reduced OS disk shedding in *rab28* mutant zebrafish, we assessed whether OS shedding was disrupted in eGFP-Rab28 transgenic fish. We used the fluorescence signal of eGFP-Rab28 itself to identify phagosomes, as it is contained in shed OS tips. Surprisingly, all three transgenic models displayed normal levels of shedding (Figures 7B–D). Although there was a slight reduction in eGFP-Rab28^WT^ retinas, this was not statistically significant (Figure 7E).

### Rab28 Biochemically Interacts With Phototransduction Proteins

**FIGURE 8:**
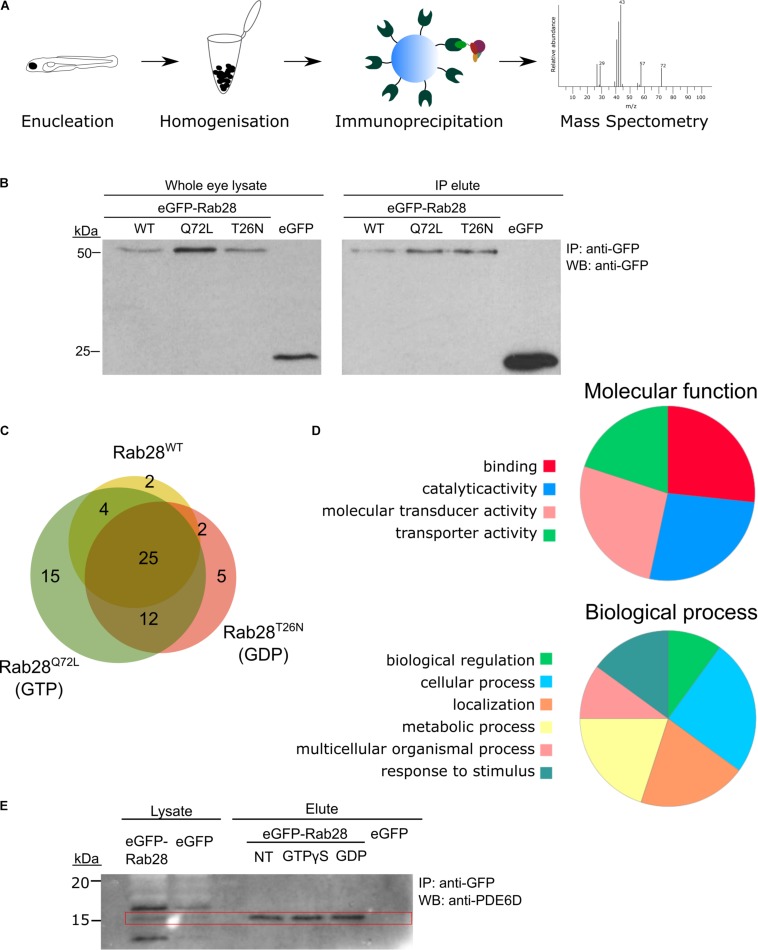
Rab28 interacts with multiple phototransduction proteins in the zebrafish eye. **(A)** Schematic of experimental workflow for Co-IP/MS of eGFP-Rab28 **(B)** Western blotting with an anti-GFP antibody, showing eGFP-Rab28 in whole eye lysate and elute after immunoprecipitation with anti-GFP beads. An untagged eGFP only control is also shown. **(C)** Venn diagram showing the number and overlap of significantly enriched (vs. GFP-only control) interacting proteins identified for each variant. **(D)** Pie charts showing gene ontology terms for proteins identified by mass-spectrometry following co-immunoprecipitation, which demonstrate a statistically significant interaction with any of the three variants. **(E)** Western blot of whole adult zebrafish eye lysate with an anti-PDE6D antibody, before and after IP of eGFP-Rab28. PDE6D band at 15 kDa mark (PDE6D mass: 17.4 kDa). Extra bands in the lysates are either post-translationally modified PDE6D, degradation products or non-specific binding by the antibody. IPs either received no treatment, GTPγS or GDP. eGFP only control is also shown.

In order to identify effectors and/or regulators of Rab28, immunoprecipitation (IP) of eGFP-Rab28 in 5 dpf zebrafish whole-eye lysates was performed, followed by mass spectrometry (Figure 8A). This was performed with eGFP-Rab28 WT, Q72L and T26N mutant lines, to identify interactants specific to the GTP and GDP-bound states. We initially tested our ability to detect and pulldown eGFP-Rab28 from larval eye extracts by immunoblot (Figure 8B), in order to estimate the number of eyes required for mass spectrometry. Using the IP-MS approach, we identified 323 unique proteins across all three eGFP-Rab28 variants, of which 52 were deemed significantly enriched (*t*-test, *p* < 0.05) (Figure 8C, Table 2 and Supplementary Table 1). The identified proteins can be divided into two groups based on fold change. The first group of 19 proteins have a log_2_ fold change > 20 for at least one of the Rab28 variants, while the second group of 33 proteins have a log_2_ fold change < 5 (Table 2 and Supplementary Table 1), and cluster accordingly (Supplementary Figure 2). To functionally categorize the Rab28 interactome, enriched gene ontology terms were identified using PANTHER-DB (Figure 8D). For the most enriched proteins across all three Rab28 variants, overrepresented processes and functions include signal transduction, cellular transport, metabolic processes, and stimulus response (Figure 8D).

**TABLE 2:** Selected interactors of RAB28 in the zebrafish eye.

Protein name	Gene name	UniProt ID	Rab28^WT^ fold change	Rab28^Q72L^ fold change	Rab28^T26N^ fold change	Statistical significance
Phosphodiesterase subunit 6δ	*pde6d*	F1QZ52	27.3322	28.9509	25.0361	
Green-sensitive opsin-1/2	*opn1mw1; opn1mw2*	Q9W6A5; Q8AYM8	27.1691	27.4838	26.0533	
Phosphodiesterase subunit 6C	*pde6c*	A0A0R4IUY2	26.6219	26.6937	27.1257	WT Q72L T26N
Mitochondrial 2-oxoglutarate/malate carrier protein	*slc25a11*	F1R319	26.3657	26.0985	26.5398	
ATP synthase peripheral stalk-membrane subunit b	*atp5f1*	B8JIS1	25.3337	25.0249	24.7418	
Synaptic vesicle glycoprotein 2A	*sv2a*	E7F6Z2	24.93	24.6194	25.0224	

Opsin-1, short-wave-sensitive 2	*opn1sw2*	Q9W6A8	18.2745	27.8295	27.0179	
Protein SREK1IP1	*srek1ip1*	Q3B7G7	15.9747	24.5876	24.3266	
Retinal G-protein coupled receptor a	*rgra*	Q567Y2	16.3406	24.2236	23.4819	
Thioredoxin	*zgc:56493*	Q7ZUI4	16.0079	23.9897	23.4402	Q72L T26N
Sideroflexin	*sfxn3*	B8JJ32	15.9322	23.9228	24.5768	
*N*-ethylmaleimide sensitive factor a/b	*nsfa/b*	B7ZV62; A0A0R4IGS4	15.5498	23.7333	23.0887	
Sodium/potassium-transporting ATPase subunit	*atp1a1; atp1a1a.4*	Q9DGL6; B8JKS7	16.4131	24.4676	24.6676	

Guanine nucleotide-binding protein beta polypeptide 3b	*gnb3b*	I3ISK4	16.1292	25.3125	16.3794	Q72L

Guanylate cyclase	*gucy2d*	F1QSL9	8.02401	16.1627	26.3006	
Erlin-1;Erlin-2	*erlin1;erlin2*	B7ZD02; F6NPB1	7.27904	7.32804	23.0652	
Cox7a2l protein	*cox7a3*	Q7SXI1	7.78141	7.69661	22.7206	T26N
Dolichyl-diphosphooligosaccharide–protein glycosyltransferase subunit 1	*rpn1*	F1QQM6; F1QTB5	15.317	14.9566	22.8419	
BetaA1c-crystallin 7	*cryba1l2*	B5M4A	14.4859	15.3575	22.151	

*Shown are those proteins with a log_2_ fold change relative to the eGFP only control ≥ 20 for at least one of the eGFP-Rab28 variants.*

There is significant overlap between the three Rab28 variants (Figure 8C), although a few proteins are enriched for one specific variant. Overall, the Rab28 interactome is highly enriched for components of the phototransduction cascade, including green and blue opsins, as well as rhodopsin, phosphodiesterase 6C, retinal guanylate cyclase and cone transducin alpha (Table 2 and Supplementary Table 1). Additionally, membrane transport proteins such as Nsfa/b (regulators of SNARE-mediated vesicle fusion), Sv2a and Erlin1/2 were significantly enriched. The identification of some non-cone proteins (e.g., Rhodopsin, Rgra) is unsurprising, as the eGFP-Rab28 bait is exposed to potential interactants from other cell types in the whole-eye lysate.

Proteins specifically enriched for particular variants include Gnb3b, which is significantly enriched for the Q72L mutant alone, while Gucy2d and Erlin1/2 are significantly enriched for the T26N mutant only (Table 2 and Supplementary Figure 2).

One protein strongly detected across all three groups was the GDI-like solubilization factor Pde6d, which is known to transport lipidated proteins to cilia and known to interact with Rab28 (Humbert et al., 2012; Ying et al., 2018). In our dataset, an equivalent fold change in Pde6d was detected across all three Rab28 groups (Table 2), although it was slightly higher for the Rab28^WT^ and Rab28^Q72L^ vs. the Rab28^T26N^ (log_2_ FC = 27.33, 28.95, and 25.04, respectively), suggesting that the latter has a lower affinity for Pde6d. As the T26N mutation lowers the affinity of Rab28 for GTP, it can be inferred that GTP-binding promotes Rab28 association with Pde6d. To test this further, IPs were performed using just eGFP-Rab28^WT^, using treatment of the lysate with an excess of either GTPγS or GDP to force Rab28 into the respective conformations. Western blots with an anti-PDE6D antibody showed co-precipitation of Pde6d with all three Rab28 baits, however, in contrast to the MS data, the amount of Pde6d pulled down was the same for GTPγS and GDP treatment and no treatment (Figure 8E). In summary, our data demonstrate that Rab28 interacts with phototransduction, and membrane transport proteins in the zebrafish larval eye.

## Discussion

### Loss of *rab28* Leads to Reduced Cone OS Shedding in Zebrafish

Mutation of *RAB28* in humans is independently linked with cone-rod dystrophy in multiple pedigrees (Roosing et al., 2013; Riveiro-Álvarez et al., 2015; Lee et al., 2017). This form of retinal degeneration is characterized by initial cone death, followed by loss of rods. In agreement, a *rab28* knockout mouse displays cone-rod dystrophy, resulting from failure of cone outer segment (COS) phagocytosis (Ying et al., 2018). In the zebrafish knockout model described here, we too find perturbed COS shedding, resulting in a significant reduction in the number of phagosome-like structures positive for COS proteins within the RPE. Our data confirms a conserved role for Rab28 in COS shedding. We also find that overexpression of Rab28 GTP/GDP-preferring mutants does not significantly alter shedding. The reason for this is unclear, though possible explanations include (i) the mutations used do not completely obliterate nucleotide exchange by Rab28 or (ii) transgene overexpression levels are insufficient to perturb OS shedding. In our previous studies in *C. elegans*, overexpression of GTP and GDP-preferring RAB-28 mutants resulted in defects in sensory compartment morphogenesis (Jensen et al., 2016), however, the degree of overexpression in that case was much higher than is achieved with our *gnat2:eGFP-rab28* reporters. We also note that the background of our transgenic lines includes WT *rab28*, which may partially ameliorate the effects of overexpression. Given its broad conservation in vertebrates and eukaryotes generally, Rab28 likely acquired this function early in vertebrate evolution, possibly arising from a general role in the shedding of membrane (in the form of extracellular vesicles) from cilia (Jensen et al., 2016; Akella et al., 2020). The recent discovery that ectosome release is a conserved feature of cilia in many different cell-types and species provides an exciting opportunity to discover further regulators of OS shedding and phagocytosis. Indeed, one can speculate that photoreceptor outer segment phagocytosis is a specialized form of ciliary ectocytosis (Carter and Blacque, 2019; Nachury and Mick, 2019), as is the case for disk morphogenesis in mouse rods (Salinas et al., 2017). It cannot be ruled out, however, that the shedding deficit observed in *rab28* null zebrafish and mice arise from defects in the RPE, rather than cones. The RPE is essential for disk shedding (Williams and Fisher, 1987) and appears to actively participate in it. If loss of Rab28 does lead to dysfunction within the RPE, however, this raises the question of why only cones are directly affected.

### *rab28* Mutant Zebrafish Have Normal Vision and Retinal Structure Up to 12 mpf

In contrast to the mouse *rab28* knockout, *rab28* knockout zebrafish display decreased RPE phagosomes, but normal visual function up to 21 dpf and no retinal degeneration up to 12 mpf. One possibility is that the level of outer segment shedding/phagocytosis remaining in zebrafish *rab28* mutants (∼40–50% of WT levels) is sufficient to support photoreceptor survival. Notably, the reduction in phagosome density in the retinas of *rab28* KO mice is higher, at approximately 80%. Zebrafish Rab28 may therefore be less essential for outer segment shedding than its mammalian orthologs.

Alternatively, genetic lesions which induce nonsense-mediated decay of mRNA were recently demonstrated to elicit a compensatory transcriptional response, whereby genes with similar functions are upregulated, masking the effect of the mutant gene (Rossi et al., 2015; El-Brolosy et al., 2019). This is particularly noted in zebrafish, where mutant phenotypes are often less severe or different from those of morpholino knockdown models (Kok et al., 2015), which do not display such compensation (Rossi et al., 2015). The absence of retinal degeneration in *rab28* knockout zebrafish may be the result of this compensatory transcriptional adaptation. Finally, there exists a high degree of redundancy in the zebrafish genome due to genome duplication thought to have occurred in the ancestor of teleost fish (Meyer and Van de Peer, 2005). However, there is no indication for a *rab28* paralog in zebrafish. More globally, functional redundancy between different Rab family members may overcome loss of *rab28* (Pavlos and Jahn, 2011; Blacque et al., 2018).

Another explanation is species differences in growth and regeneration. Unlike mammals, the zebrafish retina displays persistent neurogenesis throughout life, generating new cone photoreceptors at the periphery (Otteson and Hitchcock, 2003). Retinal injury can also elicit a response from zebrafish Müller glia, which proliferate and re-differentiate to replace lost retinal cells (Yurco and Cameron, 2005). These physiological differences may mask slow-onset thinning of the retina during degeneration.

### Rab28 Localization in Cones

Here, GFP-tagged Rab28 was highly enriched in the OS of zebrafish cones, regardless of whether it is in the GTP or GDP-bound state, suggesting all of its activity occurs within the OS, or that nucleotide binding is not important for Rab28 localization and/or function. The former scenario is in agreement with previous findings that mouse Rab28 regulates shedding from cone OS tips (Ying et al., 2018), and our own data suggesting the same. The latter case is supported by the relatively mild effects of overexpressing GTP/GDP-preferring mutants of Rab28 and our observation that the nucleotide bound state of Rab28 does not affect interaction with PDE6D, a critical transport regulator of Rab28 (Humbert et al., 2012; Ying et al., 2018). These results are surprising, given the substantial effects of GTP/GDP-binding on both the localization and function of *C. elegans* RAB-28 that we have previously demonstrated (Jensen et al., 2016; Akella et al., 2020). Our zebrafish data point to the possibility that vertebrate Rab28 is a non-canonical Rab in its behavior and function.

We also observed a striking pattern of localization with COS, particularly those of short single (SS) cones, involving the concentration of Rab28 into discrete bands throughout the OS. This banding pattern was more extensive for the Rab28^T26N^ mutant, suggesting that GDP-bound or nucleotide free Rab28 is more efficiently targeted to these sites. As the average distance between cone OS lamellae [9–13 nm (Nilsson, 1965)] is much too small to be resolved by fluorescence microscopy, these aggregations of Rab28 must be present on only a subset of lamellae within the OS. In rods and cones, a relatively consistent number of disks/lamellae are shed each time (Kocaoglu et al., 2016; Campbell and Jensen, 2017). The precise reason for the *rab28* pattern in cones and whether this has a functional purpose are fascinating future research questions. One possibility is they mark sites of contact between the OS and the RPE. Rab28 may recruit effectors to these outer segment membranes which cooperate with other proteins in the RPE membrane to initiate outer segment shedding. Why this pattern is primarily observed in SS cones may be due to a need for higher disk turnover in UV COS, arising from their absorbance of highly phototoxic UV light. At the very least, our Rab28 localization data indicates that there are differences in COS organization between different classes of cone.

Discrete banding patterns within outer segments were previously reported, though exclusively in rods (Haeri et al., 2013; Hsu et al., 2015), where it is thought to be a product of light-induced fluctuations in protein or disk synthesis. There is, however, some disagreement over whether such patterns have functional significance or are merely artifacts. Discrete patterns of localization within cone OS are themselves surprising, given that proteins can freely diffuse both laterally and axially within them, via the ciliary facing membrane which connects lamellae (Young, 1969; Bok and Young, 1972; Liebman, 1975; Willoughby and Jensen, 2012). This is in contrast to ROS, where the separation of disks precludes diffusion of membrane proteins between them. Indeed, it has been observed that a fluorescent reporter which can label discrete stacks of disks in rods becomes diffuse when expressed in COS (Willoughby and Jensen, 2012). Proteins which form banding patterns of localization in COS, such as Rab28, must therefore be prevented from undergoing diffusion and tethered to particular membranes.

One implication from this is that the oldest and most photo-oxidatively damaged proteins are evenly distributed throughout COS, while they are restricted to the tip of ROS. Thus, OS renewal is likely less efficient in cones than rods, potentially explaining why loss of *rab28* appears to exclusively affect cones and not rods.

### Rab28 Interacts With the Phototransduction Machinery of Zebrafish Cones

Using an IP-MS approach, we identified novel interactants of Rab28 in the zebrafish eye. Of these, the most notable include components of the phototransduction cascade (opsins, phosphodiesterase 6C, retinal guanylate cyclase, guanine-nucleotide binding protein 3b) and vesicular trafficking proteins (Sv2a, Nsfa/b). We also validate a previously identified interaction with the prenyl-binding protein Pde6d. Interactions with phototransduction proteins may point to a role for Rab28 in the transport of these proteins, perhaps within the OS itself, given that Rab28 almost exclusively localizes therein. The diversity of interactants either suggests roles for these proteins in photoreceptor OS, or roles for Rab28 outside the OS, such as in the inner segment (the location of photoreceptor mitochondria) or synapse.

There is a notably low degree of overlap between the interactants identified in our study and those in a previous study (Ying et al., 2018). There are several possible explanations for these discrepancies, first among them the characteristics of the species from which tissue was derived. The larval zebrafish retina is cone-dominant, consisting of ∼92% cones (Zimmermann et al., 2018), in contrast to the rod-dominant bovine retina (Szél et al., 1996) used by Ying et al. (2018), Our dataset may therefore be more enriched for the cone-specific interactome of Rab28, as suggested by the prevalence of cone-specific proteins in our interactant list. Additionally, our experiments were performed with still-developing larval tissue. We show here that retinal development is accompanied by changes in Rab28 localization within the COS and it is conceivable that this is accompanied by changes to the Rab28 interactome. Our data also offers insight into the effect of nucleotide binding on Rab28 interactions, as a small number of interactants only displayed significant interaction with one of the nucleotide binding mutants. For example, Gnb3b was significantly enriched by the Q72L (GTP-preferring) bait only, while Gucy2d and Erlin-1/2 were enriched for the T26N (GDP-preferring) mutant. Gnb3b may be a direct effector of Rab28 in its active state, while Erlin-1/2 may be a guanine nucleotide exchange factor (GEF), which promotes the exchange of GDP for GTP (Lee et al., 2009).

## Data Availability Statement

The proteomics dataset generated for this study can be found in the PRIDE database (http://www.ebi.ac.uk/pride), with the identifier PXD017523.

## Ethics Statement

The animal study was reviewed and approved by UCD Animal Research Ethics Committee and the Health Products Regulatory Authority (Project authorization AE18982/P062).

## Author Contributions

SC, OB, and BK conceived and designed experiments. SC and AM performed experiments and analyzed the data. DM contributed to design and execution of IP-MS experiments. GM contributed to design and execution of high resolution fluorescence imaging experiments. SC and BK wrote the manuscript.

## Conflict of Interest

The authors declare that the research was conducted in the absence of any commercial or financial relationships that could be construed as a potential conflict of interest. The reviewer XS declared a past co-authorship with one of the authors BK.
