# Supplementary figures and images for: Genetic Deletion of Zebrafish Rab28 Causes Defective Outer Segment Shedding, but Not Retinal Degeneration
Source: Front Cell Dev Biol. 2020 Mar 17;8:136. doi: 10.3389/fcell.2020.00136 (PMC7092623; doi:10.3389/fcell.2020.00136)

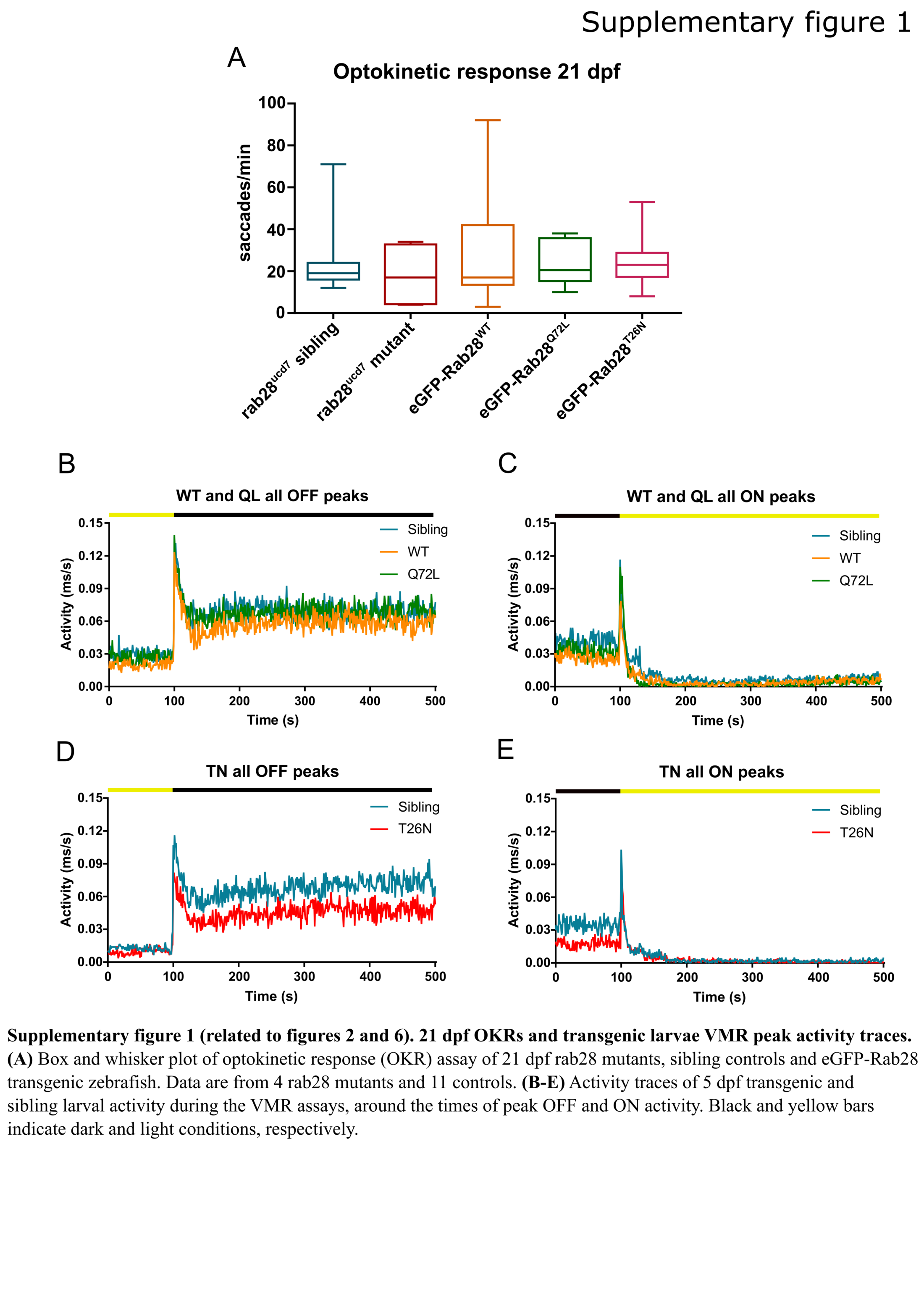

Supplement: Supplementary file 1 [file Image_1.TIF]

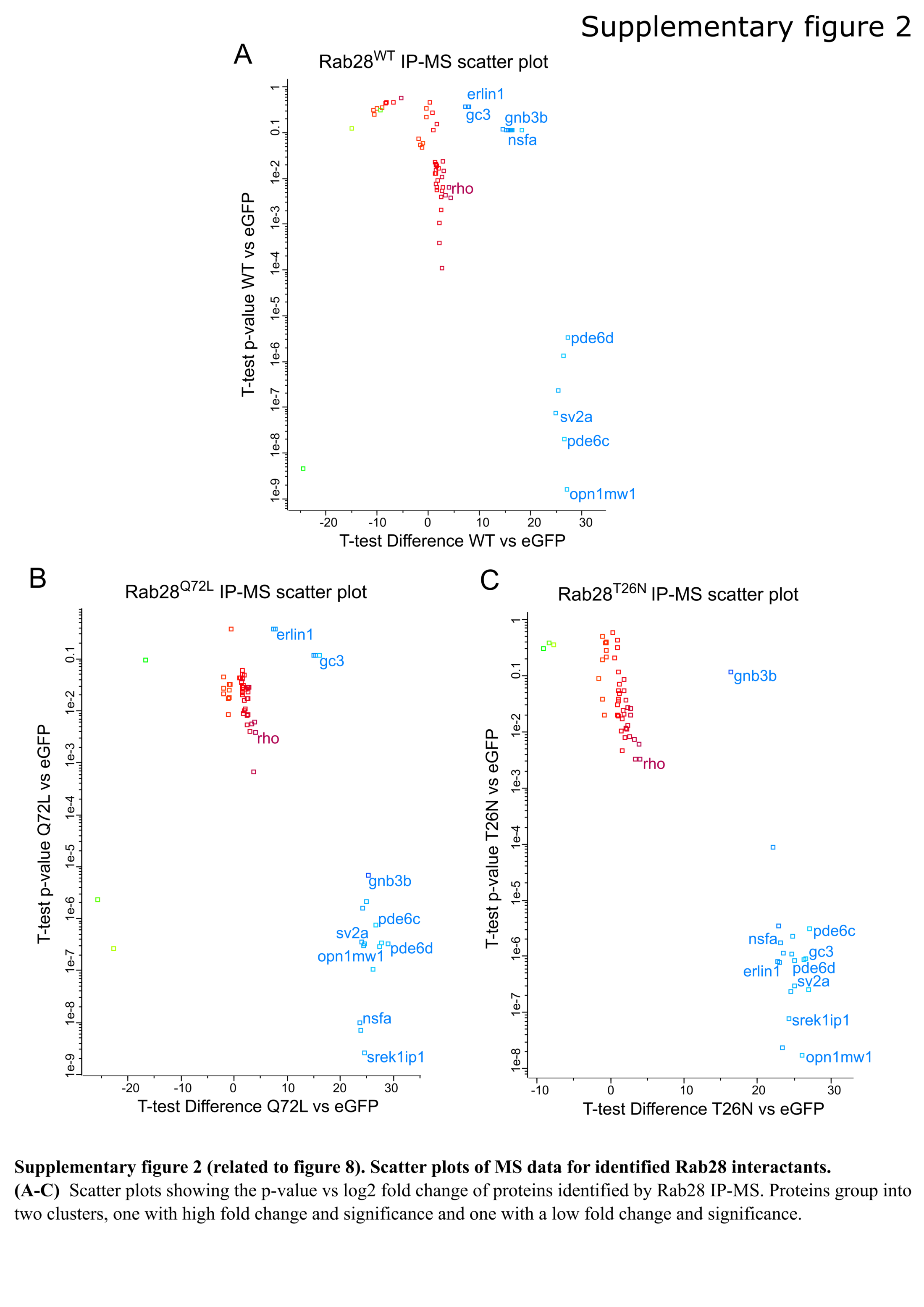

Supplement: Supplementary file 2 [file Image_2.TIF]
